# 

**Published:** 1902-12-15

**Authors:** 


### CONTENTS.

Preventive Dentistry, - - *By C. M. Wright, D.D.S.* 595

Some Things the General Practitioner ought to Take into Account in Orthodontia, *By C. B. Blackmarr, D.D.S.* 599

Dental Instruments, - - *By J. A. Watling, D.D.S.* 607

Discussion on High and Low-fusing Porcelains, *By the Odontographic Society of Chicago* 611

Editorial, - - - - - - - - - - 629

Announcements, - - - - - - - 631

Obituary, - - - - - - - - - - 632

Indents, - - - - - - - - - 633

